# Upstream AUGs and upstream ORFs can regulate the downstream ORF in *Plasmodium falciparum*

**DOI:** 10.1186/s12936-015-1040-5

**Published:** 2015-12-21

**Authors:** Mayank Kumar, Vivek Srinivas, Swati Patankar

**Affiliations:** Department of Biosciences and Bioengineering, Indian Institute of Technology Bombay, Powai, Mumbai, 400076 India

**Keywords:** Upstream ORFs, Upstream AUGs, *Plasmodium falciparum*, *var* genes, Malaria, Gene regulation, 5′ leader, Kozak sequence

## Abstract

**Background:**

Upstream open reading frames (uORFs) and upstream AUGs (uAUGs) can regulate the translation of downstream ORFs. The AT rich genome of *Plasmodium falciparum*, due to the higher AT content of start and stop codons, has the potential to give rise to a large number of uORFs and uAUGs that may affect expression of their flanking ORFs.

**Methods:**

A bioinformatics approach was used to detect uATGs associated with different genes in the parasite. To study the effect of some of these uAUGs on the expression of the downstream ORF, promoters and 5′ leaders containing uAUGs and uORFs were cloned upstream of a luciferase reporter gene. Luciferase assays were carried out in transient transfection experiments to assess the effects of uAUGs and mutations on reporter expression.

**Results:**

The average number of uATGs and uORFs seen in *P. falciparum* coding sequences (CDS) is expectedly high compared to other less biased genomes. Certain genes, including the *var* gene family contain the maximum number of uATGs and uORFs in the parasite. They possess ~5 times more uORFs and ~4.5 times more uAUGs within 100 bases upstream of the start codons than other CDS of the parasite. A 60 bp upstream region containing three ORFs and five ATGs from *var* gene PF3D7_0400100 and a gene of unknown function (PF3D7_0517100) when cloned upstream of the luciferase start codon, driven by the *hsp86* promoter, resulted in loss of luciferase activity. This was restored when all the ATGs present in the −60 bp were mutated to TTGs. Point mutations in the ATGs showed that even one AUG was sufficient to repress the luciferase gene.

**Conclusions:**

Overall, this work indicates that the *P. falciparum* genome has a large number of uATGs and uORFs that can repress the expression of flanking ORFs. The role of AUGs in translation initiation suggests that this repression is mediated by preventing the translation initiation complex from reaching the main AUG of the downstream ORF. How the *P. falciparum* ribosome is able to bypass these uAUGs and uORFs for highly expressed genes remains a question for future research.

**Electronic supplementary material:**

The online version of this article (doi:10.1186/s12936-015-1040-5) contains supplementary material, which is available to authorized users.

## Background

*Plasmodium falciparum* causes malaria in humans and results in around half a million deaths every year [1]. The parasite completes its life cycle in two hosts (humans and mosquitoes), which involves distinct morphological stages mediated by differentially expressed genes [2–4]. Several studies have compared mRNA and protein abundance data of asexual stages of the parasite life cycle and mRNA profiling of steady state and polysome associated mRNA. These studies found a lag between maximum mRNA expression and expression of the corresponding protein for many genes, indicating the presence of post-transcriptional gene regulation (PTGR) in the parasite. This is proposed to be an effective and rapid way of controlling gene expression [5–7]. Polysome profiling of parasite mRNA has also shown that ribosomal coverage in 5′ leaders of mRNAs is a common feature in the parasite. Also, it was found that a larger proportion of genes that show PTGR have a higher 5′ leader read coverage than genes that do not show PTGR [5, 6]. Upstream open reading frames (uORFs) have been proposed to play an important role in this phenomenon [5].

Upstream ORFs and upstream AUGs (uAUGs) have emerged as important players in PTGR in different organisms where they repress translation. Upstream ORFs are open reading frames containing a start and an in-frame stop codon present within the 5′ leader of mRNA while uAUGs are AUGs without any in-frame stop codon within this region. Bioinformatics studies have shown the presence of one or more uAUG/uORF in eukaryotic mRNAs [8, 9]. Indeed, 49 % of human transcripts and 44 % of mouse transcripts were shown to contain at least one uORF in their 5′ leaders. Also, for mammalian cells, mRNAs containing uORFs were found to have lower protein to mRNA ratio than mRNAs containing no uORF [9, 10].

Upstream AUGs and uORFs mediate their effects at the translation initiation step. In eukaryotes, translation initiation begins with loading of the small ribosomal subunit onto the 5′ cap of mRNA, followed by scanning in the 3′ direction. When the scanning ribosome encounters an AUG, the translation machinery assembles and translation begins [11]. However, the nucleotides surrounding the start codon, termed the Kozak sequence, determine the ability of AUGs to engage the scanning ribosome and begin translation [12, 13]. Because of the scanning mode of translation initiation in eukaryotes, the presence of uORFs and uAUGs can pose a challenge to the start codon if they engage the ribosome [14].

The strength of down-regulation of the main ORF by uAUGs and uORFs depends on several factors, one being the Kozak sequence. Upstream AUGs and uORFs with stronger Kozak sequences have a higher chance of engaging the ribosome and initiating translation at the expense of the downstream ORF [14]. The role of Kozak sequences in mediating the effect of uORFs was recently studied by introducing a 9 bases uORF upstream of the GFP start codon. By changing the Kozak sequences of the uORF and GFP, the authors were able to achieve relative GFP expression from 0.05 to 0.6 units [15]. In another study, effect of uAUGs on translation of the main ORF was tested by introducing synthetic 5′ leaders that contained varying numbers of uAUGs with different Kozak sequences [16].

This ability of uORFs to regulate translation has biological relevance as shown in yeast [17], *Neurospora* [18], plant cells [19], *Drosophila* [20], human cells [21] and viruses [22]. Mutations that alter uORFs have also been linked to diseases in humans [9]. A uAUG out of frame with the main AUG can also down-regulate the main ORF of embryonic proinsulin mRNA [23].

In *P. falciparum*, uORF mediated PTGR is seen for the *var2csa* gene, a member of the *var* gene family. The *var* genes code for erythrocyte membrane proteins called PfEMP1 (*Plasmodium falciparum* erythrocyte membrane protein 1) [24]. PfEMP1 proteins mediate sequestration and immune evasion of parasite infected RBCs [25, 26]. The parasite genome contains ~60 *var* genes [27] which show monoallelic expression [28]. This ensures that only one *var* gene is expressed at a time and to achieve this they are under different layers of regulation [29–33].

The VAR2CSA protein is thought to be responsible for binding of parasites to the placenta in pregnancy-associated malaria [34]. The *var2csa* transcripts are present in both pregnant and non-pregnant individuals [35, 36]. However, sera from non-pregnant individuals do not show reactivity against a laboratory strain of *P. falciparum* selected for binding with chondroitin sulfate A (CSA) while sera from malaria-infected pregnant women do [34, 37, 38]. These data indicate that while transcripts are expressed, the protein is present only during pregnancy suggesting translational regulation of *var2csa*.

Interestingly, a 364 bases upstream ORF (uORF) located in the 5′ leader of the *var2csa* mRNA has been shown to down-regulate the translation of a reporter gene in laboratory strains [39, 40]. Polysome profiling of in vitro grown parasites found a high ribosome density at two uORFs of *var2csa* mRNA, one of which is the 364 bases uORF [6]. This indicated that the uORFs were able to engage the scanning ribosome and initiate translation at the expense of the VAR2CSA protein.

The frequency of uATGs and uORFs is influenced by AT content of the genome, since start and stop codons are AT-rich [41]. The *P. falciparum* genome is one of most AT rich genomes sequenced thus far with an intergenic AT percentage of 90 % [27]. This raises questions about the frequencies of uORFs and uATGs in *P. falciparum* and their effects on downstream ORFs. In this report the *Plasmodium* genome was found to contain a higher number of uATGs in 5ʹ leader sequences than other organisms, with *var* genes containing the maximum number of uATGs among *P. falciparum* coding sequences (CDS). Testing the effect of uAUGs from two genes (*var* gene PF3D7_0400100 and gene of unknown function PF3D7_0517100) on parasite gene expression, using a heterologous promoter, revealed that uAUGs were able to repress the expression of a luciferase reporter gene. As all these AUGs had different Kozak sequences, the effect of 21 different Kozak sequences on reporter expression was tested and it was found that the frequency of the Kozak sequence in the genome showed no correlation with its ability to drive reporter activity. This study shows that the presence of uAUGs is a common feature of parasite mRNAs and these uAUGs can down-regulate the main ORF. This work opens up future avenues of research regarding the mechanisms used by the parasite translation machinery to bypass these uAUGs and express abundant proteins.

## Methods

### Culturing of parasites

*Plasmodium falciparum* 3D7 strain was cultured in vitro in RPMI 1640 (Life Technologies) supplemented with 10 % human plasma or 0.5 % albumax (Life Technologies), 48 mg L^−1^of hypoxanthine (Sigma-Aldrich), 2 mg ml^−1^of sodium bicarbonate (Sigma-Aldrich) and 2 mg ml^−1^ glucose (Sigma-Aldrich) containing 50 µg ml^−1^ of gentamycin (Abbott). A haematocrit of 3 % was maintained using human RBCs. Parasites were synchronized with 5 % sorbitol whenever necessary. Fresh RBCs were collected from volunteers after approval from the Institute Ethics Committee of IIT Bombay.

### Extraction of uATGs from DNA sequences

A Python script was written for extracting uATGs from 3D7 genome sequence downloaded from [42] and randomized sequences. The script can be found online [43].

### Cloning the −60 bp regions of *var* gene PF3D7_0400100 and gene of unknown function PF3D7_0517100

Single stranded oligonucleotides were annealed to generate the −60 bp upstream region of the *var* gene Pf3D7_0400100 and the gene PF3D7_0517100. Primers used for generating the mutant constructs are given below.

- *var* gene Pf3D7_0400100
-   5′CATGCCAAACCATGTATGCCACGATATAAACCACGTATGCATGACATCATGTAGTCGTGAACAA3′
-   5′CATGTTGTTCACGACTACATGATGTCATGCATACGTGGTTTATATCGTGGCATACATGGTTTGG3′
- Gene PF3D7_0517100
-   5′CATGTAATGGTTAAGCATCAGGTTAATTTTCCTATGTCATGTTCTTTATATGATATGCTTTAAA3ʹ
-   5′CATGTTTAAAGCATATCATATAAAGAACATGACATAGGAAAATTAACCTGATGCTTAACCATTA3ʹ

The annealed oligonucleotides were ligated to plasmid Pf86 digested (kind gift from Kevin Militello and Dyann Wirth, Harvard School of Public Health, Boston) with NcoI (Thermo Scientific). The cloning strategy involved disruption of the NcoI sites after successful ligation for ease of screening. A similar protocol was followed to make mutations in the −60 bp region cloned in the plasmid Pf86 (Pf86-60var) to generate constructs Pf86-60var(mut2) and Pf86-60var(mut 2 to 6). Primers used for generating the mutant constructs are given below.

- Pf86-60var(mut2)
-   5′CATGCCAAACCTAATATGCCACGATATAAACCACGTATGCATGACATCATGTAGTCGTGAACAA3ʹ
-   5′CATGTTGTTCACGACTACATGATGTCATGCATACGTGGTTTATATCGTGGCATATTAGGTTTGG3ʹ
- Pf86-60var(mut 2 to 6)
-   5′CATGCCAAACCTTGTTTGCCACGATATAAACCACGTTTGCTTGACATCTTGTAGTCGTGAACAA3ʹ
-   5′CATGTTGTTCACGACTACATGATGTCAAGCAAACGTGGTTTATATCGTGGCAAACAAGGTTTGG3ʹ

All clones were confirmed by sequencing.

### Site directed mutagenesis (SDM)

SDM was performed by PCR using non-overlapping forward and reverse primers, where one of the primers contained the desired mutation(s). Primers were phosphorylated by polynucleotide kinase (New England Biolabs) as per the manufacturer’s protocol. PCR was carried out by KAPA HiFi™ PCR kit (Kapa Biosystems) as per the manufacturer’s protocol. The PCR product was treated with DpnI (Thermo Scientific) to cleave the parental plasmid, after which the PCR products were ligated and transformed. SDMs were performed to generate constructs Pf86-60var(mut 1 to 6) using plasmid Pf86-60var(mut 2 to 6) as PCR template and Pf86-60var(mut1, 3, 4, 5, 6) and Pf86-60var(mut 1 to 5) using plasmid Pf86-60var(mut 1 to 6) as PCR template. Primers used for generating the mutant constructs are given below.

- Pf86-60var(mut 1 to 6)
-   5′CTCAACGGCCTTGCCAAACC3ʹ
-   5′ATTTTATTCGAAATGTGGGAAG3ʹ
- Pf86-60var(mut1, 3, 4, 5, 6)
-   5′GCCTTGCCAAACCATGTTTGCCAC3ʹ
-   5′CGTTGAGATTTTATTCGAAATGTGGG3ʹ
- Pf86-60var(mut 1 to 5)
-   5′GCTTGACATCATGTAGTCGTGAAC3ʹ
-   5′AAACGTGGTTTATATCGTGGCAAAC3ʹ

SDM was also used to mutate the ATGs present in the 60 bp upstream sequence of the gene PF3D7_0517100 cloned in the plasmid Pf86 [Pf86-60cntr mut(1 to 6)]. Primers used for this SDM were:

- 5′GTTAATTTTCCTTTGTCTTGTTCTTTATTTGATTTGCTTTAAAC3ʹ
- 5′CTGATGCTTAACCAATACAAGGCCGTTGAG3ʹ

All the clones were confirmed by sequencing.

### Cloning of the luciferase start codon with different Kozak sequences

Plasmid Pf86 was digested with BstBI (Thermo Scientific). One of the BstBI sites was present in the *hsp86* 5′ leader (at −16 bp upstream of the start codon) while the other site was present in the luciferase coding sequence (at +165 bp downstream of the start codon). The digestion of the plasmid resulted in the release of the luciferase start codon along with the Kozak sequence. To introduce different Kozak sequences, SDMs were carried out by PCR using plasmid Pf86 as template with a specific set of forward primers intended to introduce desired Kozak sequences and a common reverse primer complementary to +150 bp region described above. Names and sequences of the primers used are provided in Additional file 1: Table S1. The PCR products were then digested with BstBI and ligated to BstBI digested plasmid Pf86. Clones were confirmed by sequencing.

### Transfection and luciferase assay

Plasmids were isolated with the QIAGEN Maxi-prep kit as per the manufacturer’s protocol. Transfections were carried out using the pre-loaded RBC protocol [44]. For each transfection, 100 µg of test firefly luciferase plasmid were mixed with 100 µg of Renilla plasmid (pPfrluc) which acts as control to normalize the firefly luciferase readings. After transfection of un-infected RBCs, the RBC pellet was washed and re-suspended in 5 ml complete medium, and infected RBCs (iRBCs) at the late trophozoite stages were added to give a final parasitemia of 0.2–0.35 %. The medium was changed every 24 h and luciferase assays were performed 80–85 h post infection when most of the parasites were in late trophozoites. The parasite pellets for luciferase assays was obtained by saponin lysis of the iRBCs. The parasites were lysed by subjecting isolated parasites to three freeze–thaw cycles alternating between liquid nitrogen and 37 °C in 60 µl of 1 X Passive Lysis buffer (Promega). The cell debris was removed by centrifuging at 10,000 rpm for 1 min. The luciferase assays were carried out using the Promega Dual-luciferase kit as per the manufacturer’s protocol. The relative light units (RLUs) were measured by a luminometer (Berthold Junior LB 9509) cumulatively over 30 s or a scintillation counter (PerkinElmer, Tri-Carb^R^ 2810TR) in the single photon counting (SPC) default mode where it behaves as a luminometer. The scintillation counter gave counts per minute (CPM) after capturing the photons for 30 s. Both firefly and *Renilla* luciferase activities were measured for equal amount of time.

## Results

### Intergenic regions of the *P. falciparum* genome contain a large number of uATGs and uORFs

**Fig. 1:**
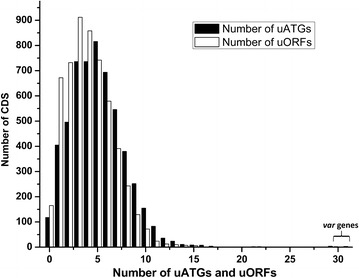
Distribution of number of uATGS and uORFs across annotated CDS of the genome of *P. falciparum.* uATGs and uORFs were calculated from 350 bp region upstream of the start codons of 5401 annotated CDS of the parasite. *var* genes contain highest number of uATGs (*highlighted*). Number of uORFs has been taken from our previous study [45]

AT-rich genomes have a high frequency of uATGs and uORFs as start and stop codons are themselves AT-rich [41]. The genome of *P. falciparum* is ~90 % AT-rich in the inter-genic region [27]. As a result, the genome is expected to have a large number of uATGs. In agreement with this, a recent report shows that the parasite genome contains a large number of uORFs [45]. A Python script was written [43] to identify uATGs within 350 bases upstream of annotated start codons since the average 5′ leader length of parasite mRNAs is predicted to be ~346 bases [46]. The program identified 27,760 uATGs which were associated with 5283 annotated CDS. This indicates that 97.8 % of the parasite CDS contain at least one uATG (Fig. 1, Additional file 2: Table S2).

Among the genes which contained a staggering number of 31 uATGs were three of the *var* genes (PF3D7_0412700, PF3D7_0412900, and PF3D7_1240600). Other genes with large numbers of uATGs were *var* gene (PF3D7_0808600), genes that code for phospholipase (PF3D7_0814400), translation initiation factor (PF3D7_1456000), plasmepsin (PF3D7_1430200), dynein (PF3D7_0927500) and some conserved proteins with unknown functions.

### *var* genes contain higher numbers of uATGs and uORFs than other genes 

Consistent with an earlier report [47], large numbers of uATGs were observed in *var* upstream regions. The *var* genes and their associated uATGs and uORFs [45] were further analysed. Each *var* gene has an average of ~7 uORFs and ~9 uATGs while other CDS contain ~4 uORFs and ~5 uATGs within 350 bases upstream of their start codons. Some *var* genes contained 29 uORFs and 31 uATGs within this length of upstream region (Fig. 1). Additionally, the average GC content of the 350 bases upstream sequences of the *var* genes was ~20 %, while that of other genes was ~13.3 %. This is in contrast with the expectation that GC rich sequences should have smaller number of uATGs and uORFs. This indicates that the large number of uATGs and uORFs found flanking *var* genes may not be entirely due to sequence composition and might be biologically relevant.

**Fig. 2:**
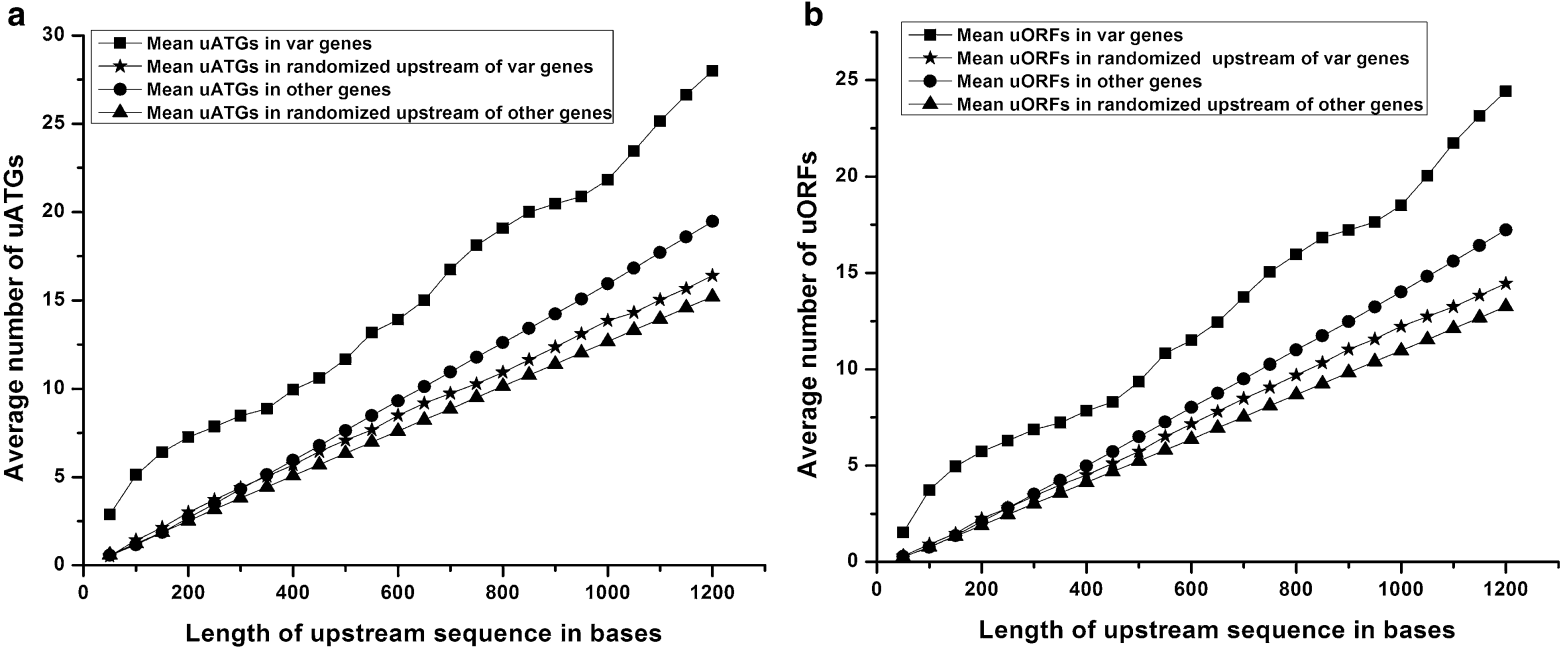
Average number of uATGs and uORFs in different protein coding genes. Average number of uATGs (**a**) and uORFs (**b**) found in the regions upstream of the start codons of *var* and other genes. Randomized upstream sequences of *var* and other genes were used as controls. Mean uATGs and uORFs were calculated in windows of 50 bases, up to 1200 base pairs upstream of the start codons

Further, relative frequencies of uATGs and uORFs were compared among the upstream regions of *var* and other genes and randomized sequences of the same. The average number of uATGs and uORFs was calculated in windows of 50 bases, up to 1200 bases upstream of the start codon. 1.2 kb was selected since the transcription start sites of three of the *var* genes have been predicted to be at around −1200 bases [31, 48, 49]. Interestingly, the average number of uATGs and uORFs proximal to *var* genes was consistently higher than those found in other genes and their randomized upstream sequences throughout the length of the upstream regions under study (Fig. 2). Higher frequencies of uATGs and uORFs in *var* 5′ upstream regions in comparison to randomized sequence also suggest that it is not a random phenomenon and may have some functional significance.

**Fig. 3:**
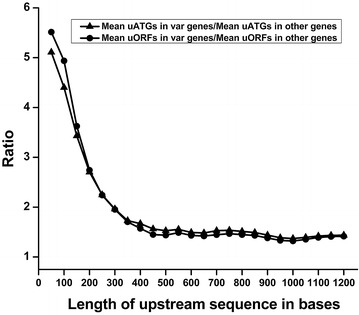
Ratio of uATGs and uORFs. Ratio of average number of uATGs and uORFs associated with *var* and other genes of the parasite with increasing length of the regions upstream of the start codons. Ratios were calculated in windows of 50 bases, up to 1200 base pairs upstream of the start codons

Also of note is that the fold differences in the average number of uATGs and uORFs associated with *var* and other genes were not uniform (Fig. 3). Within 50 bases upstream of the start codon, there were ~5 times more uATGs and uORFs in *var* genes versus other genes. This is largely due to the fact that other genes have virtually no uATGs and uORFs in these regions while *var* genes have an average number of ~1.5 uORFs and ~3 uATGs in the same region. Up to 300 bases upstream of the start codon, the fold difference between *var* gene-associated uATGs and uORFs and other genes-associated uATGs and uORFs decreases until it reaches ~2; after 300 bases the fold difference in average uATGs and uORFs levels off at ~1.5. These data indicate a high frequency of uATGs and uORFs within 100 bases of the start codon of *var* genes. Should the ribosome initiate at one of these numerous uATGs and uORFs, profound effects on translation of the downstream *var* gene could be envisaged.

### Insertion of a *var* upstream sequence represses expression of a downstream luciferase reporter gene

To study the effect of *var* gene-associated uATGs/uORFs on the translation of the downstream ORF, the −60 bp region (−60 to −1) from one of the *var* genes was cloned upstream of a luciferase reporter gene. The region was chosen because the 50 bp *var* upstream regions contain ~5 times more uORFs and uATGs than other CDS of the parasite (Fig. 3). The 60 bases *var* upstream sequence was expected to be part of the *var* mRNA since the average length of the 5′ leader sequences of the parasite mRNA is predicted to be 346 bases and the length of 5′ leader sequences of a few *var* genes is more than 1 kb [31, 48, 49]. Nevertheless, RT-PCR of *var* upstream regions carried out from parasite RNA revealed that the 60 bases *var* upstream sequence was indeed part of *var* mRNA (Additional file 3: Figure S1). Therefore, this 60 bp *var* upstream sequence was tested for its effect on the expression of a downstream ORF.

The effects of uORFs on *var* gene expression have been tested for the *var2csa* gene where a *var2csa*-uORF down-regulates the translation of a downstream ORF [39, 40]. These studies have used the approach of stable transfection of a plasmid carrying a drug resistance gene under a constitutive promoter and a reporter gene (luciferase coding sequence or drug resistance marker) driven by the *var* promoter and *var* 5′ leader. Effect of the mutations in the *var* uORF are tested by quantifying luciferase expression or by measuring the time taken for the stable line to be selected under drug pressure, when resistance marker is used.

In this report, a transient transfection approach was used to study the role of uAUGs and uORFs in regulation of the luciferase gene expression. In these experiments, the time taken for testing constructs is fast (80–85 h), however due to low transfection efficiencies, it is not possible to test whether mutations in the 5ʹ leader have any effect on mRNA levels. Therefore, care has been taken to state that the results reflect the effects of uAUGs and uORFs on “gene expression” of the luciferase reporter. Gene expression is here defined as the sum of mRNA levels and translation.

To assess the effect of *var* sequences proximal to the start codon on the expression of the downstream ORF, the −60 bp region of one of the *var* genes with five uATGs and three uORFs (PF3D7_0400100) was cloned upstream of the luciferase start codon in vector Pf86 (Pf86-60*var*). The upstream region inserted into Pf86 contained five ATGs (ATG^2^ to ATG^5^), but during cloning, an extra ATG (ATG^1^) was introduced in the vector since the upstream sequence was cloned at an NcoI site. Pf86 vector was used as positive control and the Pf86 vector that had a G to T mutation at the +4 position (resulting in generation of a stop codon immediately after the start codon of luciferase; Pf86-stop) was used as a negative control.

**Fig. 4:**
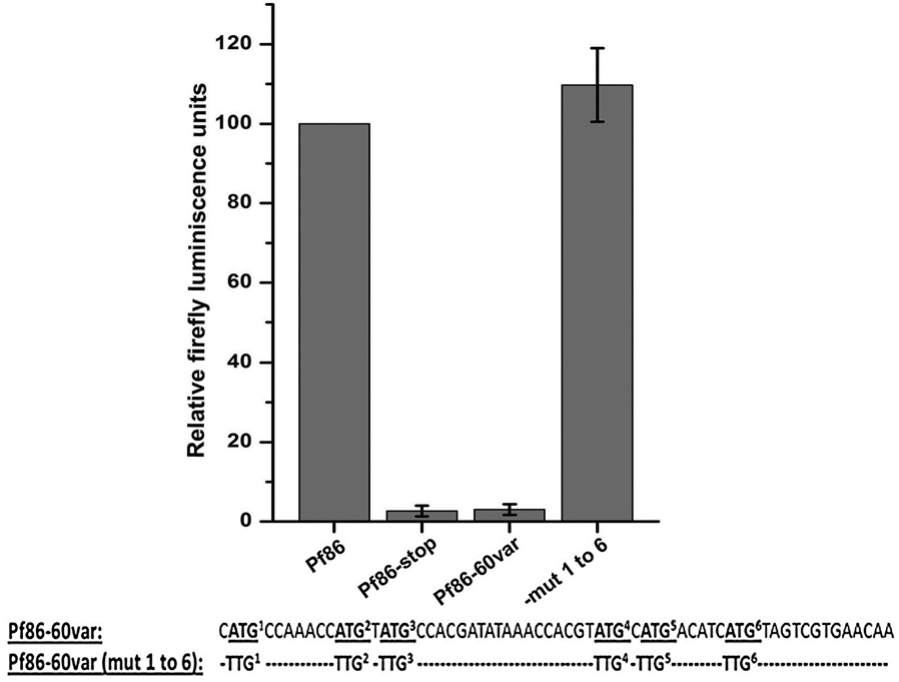
Luciferase assay of the constructs containing *var* upstream region and upstream region with mutated ATGs. Firefly luciferase readings of the constructs Pf86, Pf86-60var and Pf86-60var(mut 1 to 6) obtained after transiently transfecting them in the parasite. Construct Pf86-stop was used as a negative control where the second codon of the luciferase CDS in the vector Pf86 was converted to a stop codon. Firefly luminescence units were normalized against those of *Renilla* for each construct. Normalized Firefly readings of each constructs have been expressed with respect to normalized Firefly readings of Pf86. Firefly luciferase readings and standard deviations have been calculated from five replicates. Normalized firefly readings of Pf86 were in the range of 1000–7000 RLUs, measured in a luminometer

The construct Pf86-60*var* was transiently transfected and luciferase assays performed. The introduction of 60 bases of *var* upstream sequences resulted in a loss of luciferase activity to the level of the negative control (Fig. 4). To check the role of uAUGs in this phenomenon, a construct where all the six uATGs were mutated to TTG [Pf86-60var(mut 1 to 6)] was generated. Luciferase readings of the construct Pf86-60var(mut 1 to 6) were found to be restored to the level of the positive control Pf86 (Fig. 4). This result indicated that the uAUGs present in the 60 bp region are capable of repressing the expression of the luciferase gene.

### A single uAUG is sufficient to repress expression of the luciferase gene

Further, to test the strength of individual uAUGs in repressing expression of the luciferase gene, constructs were generated to contain only one uATG at a time. Constructs Pf86-60var(mut 2 to 6), Pf86-60var(mut 1, 3, 4, 5, 6) and Pf86-60var(mut 1 to 5) had all the ATGs in the 60 bp region mutated to TTG except ATG^1^, ATG^2^ and ATG^6^ respectively. Construct Pf86-60var(mut 2) was also generated in which only ATG^2^ was mutated.

**Fig. 5:**
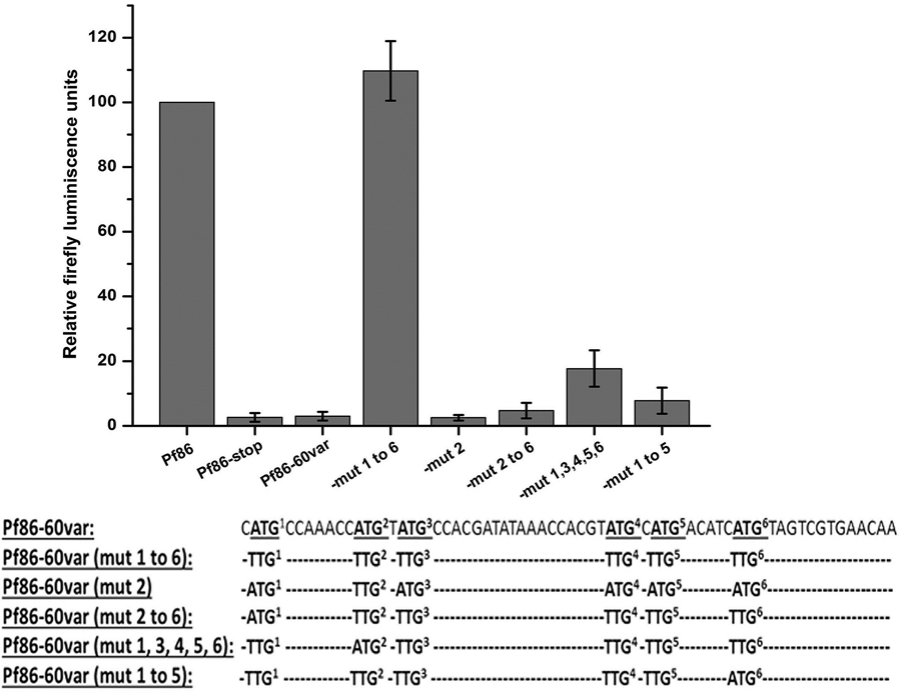
Firefly luciferase readings of the constructs Pf86 and Pf86-60var containing different point mutations. Firefly luciferase readings of the constructs Pf86, Pf86-60var and Pf86-60var(mut 1 to 6), Pf86-60var(mut 2), Pf86-60var(mut 2 to 6), Pf86-60var(mut 1, 3, 4, 5, 6)_,_ and Pf86-60var(mut 1 to 5) obtained after transiently transfecting them in parasite. Construct Pf86-stop where the second codon of the luciferase gene in the vector Pf86 was converted to a stop codon was used as a negative control. Firefly luminescence units were normalized against those of *Renilla* for each construct. Normalized Firefly readings of each construct have been expressed with respect to normalized Firefly readings of Pf86. Firefly luciferase readings and standard deviations have been calculated from five replicates. Normalized firefly readings were in the range of 1000–7000 RLUs, measured in a luminometer

Luciferase activities of the constructs were measured after transient transfections. The expression levels of the constructs were similar to the negative control indicating that even one uAUG is sufficient in repressing expression of the downstream ORF (Fig. 5). However, individual uAUGs showed varied levels of repression: construct Pf86-60var(mut 2 to 6) showed ~5 % of Pf86 luciferase activity, while constructs Pf86-60var(mut 1 to 5) and Pf86-60var(mut 1, 3, 4, 5, 6) showed ~10 and ~20 % of Pf86 luciferase activity respectively (Fig. 5).

The loss of luciferase activities of all these constructs appears to be due to the uATG having an in frame stop codon and leading to the formation of an ORF. The uATG1 initiates an ORF of 147 bases that overlaps with the luciferase ORF, while uATG2 initiates a uORF of 18 bases. The uATG6 is immediately followed by a stop codon. Thus, the absence of luciferase activity of the constructs appears to be due to the failure of the scanning ribosome to reach the luciferase start codon.

### Upstream sequence from a gene of unknown function also represses luciferase gene expression

**Fig. 6:**
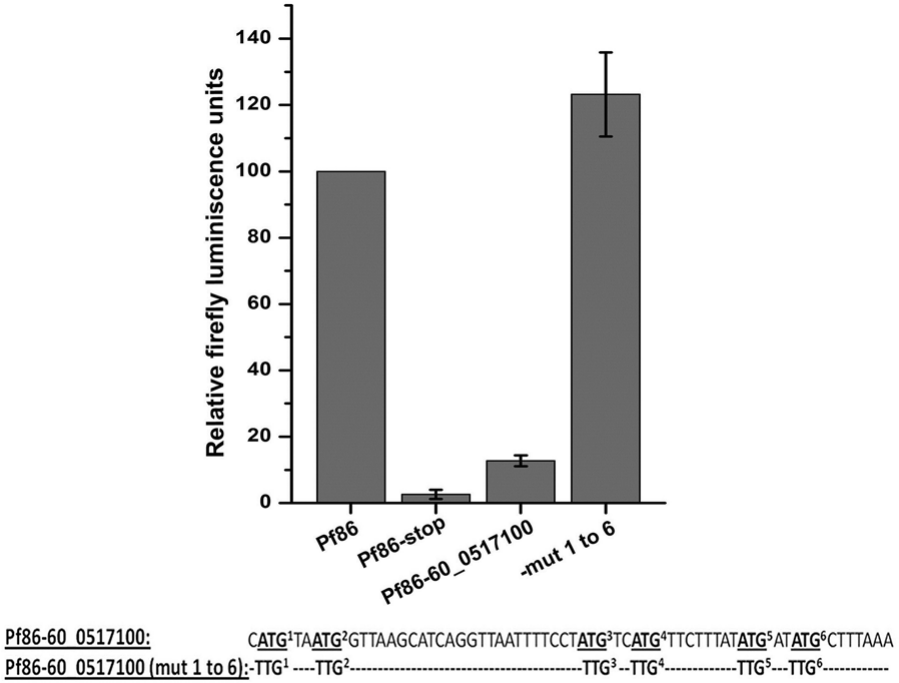
Luciferase assay of the constructs containing upstream region from a gene with unknown function and upstream region with mutated ATGs. Firefly luciferase readings of the constructs Pf86, Pf86-60_0517100 and Pf86-60_0517100(mut 1 to 6) obtained after transiently transfecting them in the parasite. Construct Pf86-stop was used as a negative control where the second codon of the luciferase CDS in the vector Pf86 was converted to a stop codon. Firefly luminescence units were normalized against those of *Renilla* for each construct. Normalized Firefly readings of each constructs have been expressed with respect to normalized Firefly readings of Pf86. Firefly luciferase readings and standard deviations have been calculated from four replicates. Normalized firefly readings of Pf86 were in the range of 2300–4000 RLUs, measured in a luminometer

In order to test if the ability of the uAUGs to repress the downstream ORF is specific to *var* gene upstream sequence, a −60 bp sequence was cloned from a gene of unknown function, PF3D7_0517100. The gene was selected since the 60 bp upstream sequence of this gene also contained five uATGs and three uORFs and its sequence was different from that of the *var* gene upstream sequence cloned (Pf86-60var). During cloning, an extra ATG (ATG^1^) was introduced resulting in a total of six uATGs. The clone (Pf86-60_0517100) was transiently transfected and luciferase assays were carried out. The construct resulted in only ~15 % of the luciferase activity in comparison to Pf86 (Fig. 6).

To check if the repression of the luciferase activity was mediated by uAUGs, all the six uATGs were mutated [Pf86-60_0517100(mut 1 to 6)]. The mutations restored luciferase activity indicating that the repression was mediated by uAUGs (Fig. 6). Therefore, it appears that the ability of uAUGs to repress the downstream ORF is a general phenomenon observed in *P. falciparum*.

### The ability of a Kozak sequence to repress reporter expression does not correlate with the frequency in the *P. falciparum* genome

As discussed earlier, the Kozak sequence is the most important feature governing the strength of uAUGs [15]. However, little is known about the strength of Kozak sequences in *P. falciparum*. In this report, individual uAUGs were capable of repressing the expression of luciferase gene effectively (Fig. 5). When the Kozak sequences of these uAUGs were analysed, it was observed that they were different, some of which (Kozak sequences of Pf86 and uATG1) were not found associated with any annotated parasite CDS while Kozak sequences of Pf86-60var and uATG2 were found at low frequencies. The observation that uAUGs with differing Kozak sequences could repress luciferase led us to check whether Kozak sequences frequently found in the parasite genome are able to drive higher reporter expression.

For this, the frequencies of Kozak sequences associated with 5401 annotated CDS of *P. falciparum* were calculated. The −5 to +4 positions of annotated start codons were considered since a bioinformatics study [45] indicated these positions to be important in deciding the translational ability of a start codon of mRNA. Out of 4096 possible Kozak sequences, the parasite uses 1086 Kozak sequences (Additional file 4: Table S3). As expected due to the AT biased genome, AAAAAatgA was the most frequent Kozak sequence, (318) followed by TAAAAatgA (151).

In mammals, frequent Kozak sequences are found to be stronger in driving translation [50]. Therefore, the relationship between the frequency and the ability of Kozak sequences to drive reporter expression in *P. falciparum* was tested. Due to lack of mRNA data, only the quantitative effect of a particular Kozak sequence on reporter gene expression has been tested. For this, 21 of the Kozak sequences of different frequencies from the most frequent (318) to least frequent (0) sequence were selected.

**Fig. 7:**
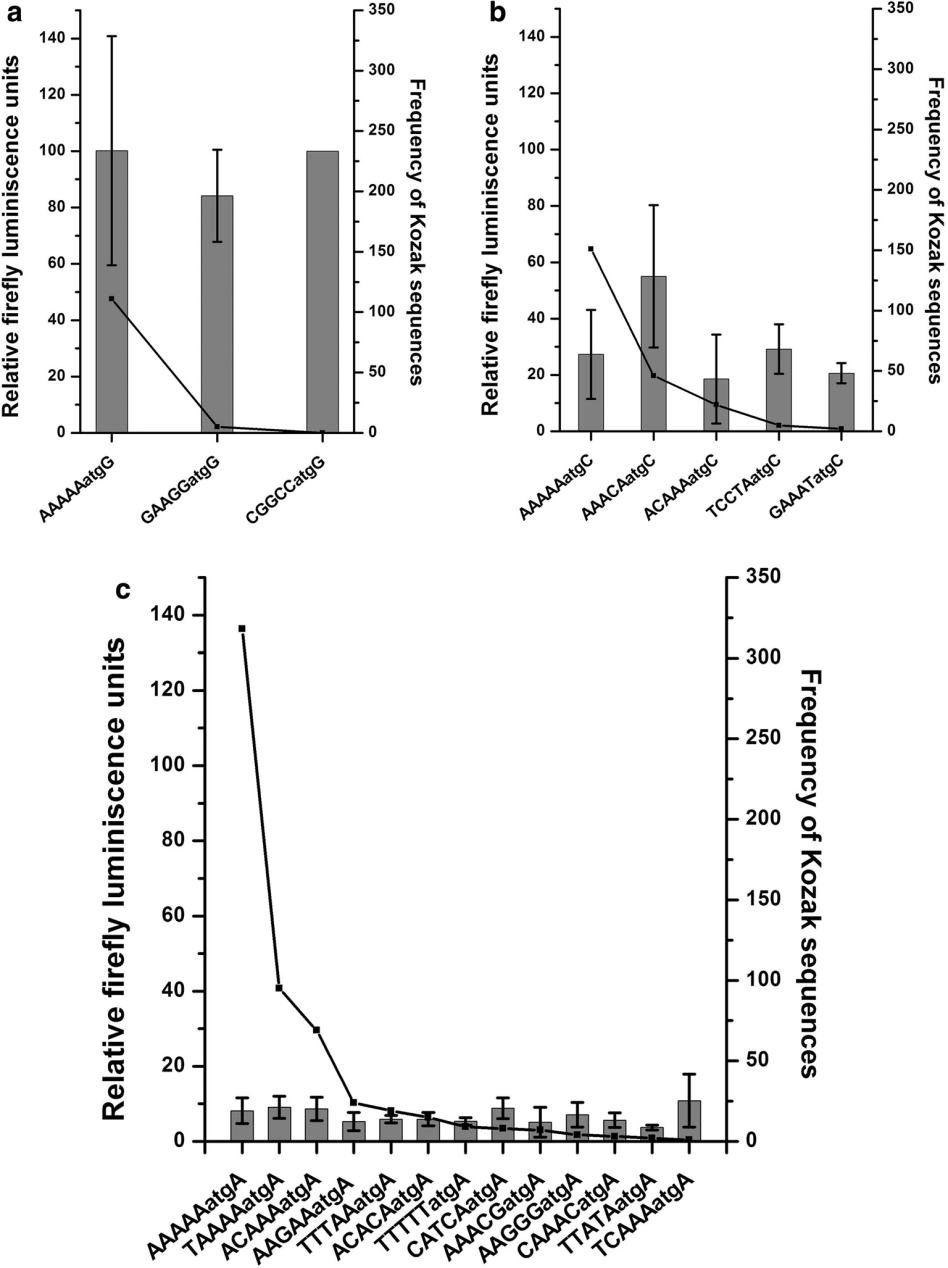
Firefly luciferase readings of the plasmid Pf86 and its derivatives under different Kozak sequences with their frequencies. Firefly luciferase readings of the plasmid Pf86 and its derivatives carrying luciferase start codon under different Kozak sequences have been shown (*bar graph*). Frequency of occurrence of different Kozak sequences in the genome tested for their strength has been shown on secondary Y-axis (*line*). Firefly luminescence units were normalized against those of *Renilla* for each constructs. Normalized Firefly readings of each constructs have been expressed with respect to normalized Firefly readings of Pf86. Luciferase readings have been measured in a scintillation counter. Firefly luciferase readings and standard deviations have been calculated from four replicates. Normalized Firefly readings were in the range of ~800,000 to ~28,00,000 CPM, measured using a scintillation counter. Constructs containing different Kozak sequences were grouped into three categories based on the +4 position: those containing ‘G’ at +4 position (**a**), ‘C’ at +4 position (**b**) and ‘A’ at +4 position (**c**)

The plasmid Pf86 was modified such that the luciferase start codon was surrounded by different Kozak sequences. Thus, 20 constructs were generated. Wild type Pf86 was taken as a control. No correlation was observed between the reporter activity and the frequency of the Kozak sequences in *P. falciparum*, unlike in other eukaryotes. In fact, the nucleotides (−5 to −1) preceding the start codon showed scant effect on expression of the luciferase gene (Fig. 7). To reach this conclusion, the constructs were classified based on the nucleotide present at the +4 position as this was within the luciferase coding region and would alter the second amino acid of the luciferase protein. Comparisons were made between constructs having the same nucleotide at the +4 position with different nucleotides at the −5 to −1 position.

Constructs containing ‘G’ at +4 positions showed high firefly luciferase activities (~100 %) despite containing very different bases at positions −1 to −5 (Fig. 7a). Similarly, for constructs containing ‘C’ at +4 positions, luciferase activities remained around 20–30 % except one even after altering the preceding bases completely (Fig. 7b). Finally, low firefly activities (~10 %) were obtained from all the constructs containing different Kozak sequences at −1 to −5 positions but ‘A’ at +4 positions (Fig. 7c). Therefore, it appears that nucleotides preceding the start codon (−5 to −1) do not play a significant role in determining the strength of the Kozak sequences. As a result the Kozak sequence which was found to be one of the strongest (CGGCCatgG) was not present in the parasite genome at all (Fig. 7).

## Discussion

This report adds to the accumulating data on the effect of uAUGs and uORFs on the expression of the downstream ORF in *P. falciparum*.

### Upstream AUGs are likely to regulate downstream ORFs via translation

In this report, the mRNA levels of different constructs in the transfection experiments have not been verified. Therefore, all statements have been made with regard to the effects of uAUGs and uORFs on the “expression” of the luciferase reporter, where expression is defined as the sum of mRNA and protein levels. However, the possibility of mRNA instability by introducing various point mutations in different constructs (Fig. 5) cannot be ruled out in the current study. Nevertheless, translational repression might be the mechanism of uAUG action because of following reasons. It is unlikely that changing single A to U at different positions in the 5′ leader plasmid Pf86-60var (Fig. 5) would affect the stability of mRNAs. There are several reports where point mutations in uAUGs have been shown to de-repress the translation of downstream ORF without altering the mRNA levels [9, 20, 40, 51–56]. As indirect evidence, the stability of the −60 bp region was predicted by the Mfold algorithm [57] and mutating the uAUGs does not alter the stability of the −60 bases sequences significantly (ΔG of the most stable folding ranged between −14 and −9.00 kcal/mol). Finally, evidence supportive of the contention that mutations may not affect mRNA stability comes from our experiments where another set of mutations, altering uATGs to TTGs completely restores luciferase activity (Fig. 6), strongly indicating that the underlying phenomenon is translation regulation.

The effect of uAUGs on translation is mediated either in a peptide-dependent or peptide-independent manner. In the peptide-dependent mechanism, a peptide formed after the ribosome begins translation at the uAUG binds the ribosome and stalls it [58]. There are only few reports of uAUGs acting via a peptide-dependent mechanism [58, 59]. In the peptide-independent mechanism, when a scanning ribosome encounters a uAUG, it can begin translation resulting in down-regulation of the main ORF or can simply continue scanning and initiate translation from the next AUG in a favorable context [60]. In such cases, down-regulation of the main ORF takes place because of the reduced probability of the scanning ribosome reaching the main AUG [61] or due to the presence of rare codons in the region between uAUG and main AUG [52] or due to the reduced concentration of translation factors [61].

### *P.falciparum* parasites might employ uAUGs to regulate the translation of mRNAs

*hsp86* is a house-keeping gene and codes for a heat shock protein. The 5′ leader of *hsp86* is 686 bases long and contains a single uAUG 495 bases upstream of the start codon. Since uAUG close to the mRNA cap is less efficient in repressing translation [62], this *hsp86* uAUG may not affect translation of the downstream ORF. On the other hand, *var* genes contain on an average of ~5 uATGs within 50 bases upstream of the start codon. When 60 bases upstream region of a *var* gene and a gene with unknown function containing five uATGs was introduced within the 5′ leader of *hsp86,* luciferase activity was completely lost (Figs. 5, 6), hinting at the role of uAUGs in translational efficiency and protein abundance.

For mammalian cells, mRNAs containing uORFs were found to have lower protein to mRNA ratio than mRNAs containing no uORF [9, 10]. This indicates that cells might use different numbers of uATGs/uORFs to fine tune the translation of a mRNA with genes encoding low abundance proteins having higher numbers of uAUGs/uORFs. Hints that *P. falciparum* might use this strategy come from our data showing that parasite CDS have different numbers of uATGs/uORFs (Fig. 1) and that insertion of six uAUGs into a heterologous promoter drastically reduces luciferase activity (Figs. 5, 6). To further explore this, a preliminary analysis of polysome profiling data [5] was conducted by linking genes having exon reads of polysome-bound mRNAs with their number of uATGs (data from this report). This analysis revealed a weak negative correlation between the number of uAUGs and the raw exon reads of the CDS; mRNAs with higher read counts in their CDS are likely to contain lesser number of uAUGs/uORFs. Assuming that mRNAs with higher reads in the CDS are likely to have higher translational efficiency, this preliminary analysis hints at a possible correlation between the number of uAUGs and protein levels. However, the hypothesis needs to be tested further by quantifying parasite proteins whose genes contain different numbers of uAUGs/uORFs.

### Large numbers of uAUGs in parasite mRNAs will pose challenge for the translation initiation machinery scanning the leader

**Fig. 8:**
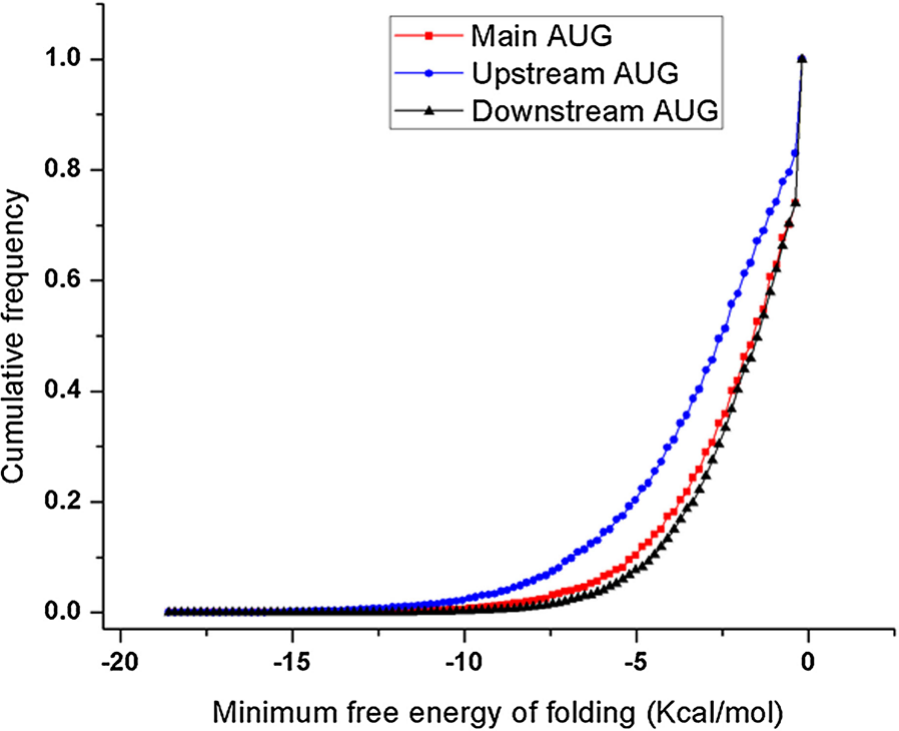
Folding energy of regions surrounding AUGs. The cumulative frequency graph of minimum folding energy, ΔG (Kcal/mol) of regions around AUGs (−15 bases to +20 bases). AUGs were characterized based on their positions in the mRNA: upstream, downstream and main AUGs

It appears that the nucleotides preceding the start codon do not play a large role in determining the strength of a Kozak sequence (Fig. 7). This is consistent with our data where uAUGs having different Kozak sequences were all able to repress the luciferase gene very strongly (Fig. 5). This leads to the next intriguing question: how is the translation of *var* and other mRNAs containing large number of uAUGs achieved? One interesting way to achieve this is by ribosome shunting, where a scanning ribosome skips a part of the 5′ leader of mRNA. Recently, ribosome shunting has been shown to skip a uAUG with a favourable Kozak sequence, and also a potential hairpin loop in the 5′ leader of the mRNA [63]. Interestingly, differences in the secondary structure of the region surrounding uAUGs and main AUG were also observed (Fig. 8). uAUGs were found to be present in a more stable secondary structure than main AUGs. Whether this difference is relevant for the translation machinery to skip uAUGs and recognize the main AUG remains to be investigated.

## Conclusions

uAUGs and uORFs have been shown to down regulate translation in different organisms. This report shows that *P. falciparum* CDS contain a large number of uATGs and uORFs among which *var* genes contain the maximum number. Presence of these uAUGs could pose a challenge for the scanning ribosome to locate the main AUG. Through transient transfection experiments, it has been confirmed that uORFs and uAUGs are able to down-regulate the expression of the luciferase gene. This report sets the stage for future work on the mechanisms by which uAUGs and uORFs down-regulate flanking ORFs, their potential role in mediating post transcriptional gene regulation and how the parasite translation machinery skips uAUGs and starts translation from the main AUGs.

## Additional files

10.1186/s12936-015-1040-5 List of primers used to generate Pf86 containing luciferase start codon under different Kozak sequences.
10.1186/s12936-015-1040-5 Numbers of uATGs associated with each annotated CDS in the parasite genome.
10.1186/s12936-015-1040-5 RT-PCR of the mixed stage parasite RNA.
10.1186/s12936-015-1040-5 Frequency of different Kozak sequences in annotated CDS in the parasite genome.

## Abbreviations

uAUG/ATG: upstream AUG/ATG
uORF: upstream open reading frame
CDS: coding sequences
