# Supplementary material for: Upstream AUGs and upstream ORFs can regulate the downstream ORF in Plasmodium falciparum
Source: Malar J. 2015 Dec 21;14:512. doi: 10.1186/s12936-015-1040-5 (PMC4687322; doi:10.1186/s12936-015-1040-5)
Supplement: Supplementary file 1 — 10.1186/s12936-015-1040-5 List of primers used to generate Pf86 containing luciferase start codon under different Kozak sequences. [file 12936_2015_1040_MOESM1_ESM.docx]

**Supplementary Table 1.**

List of primers used to generate Pf86 containing luciferase start codon under different Kozak sequences.

| **Name** | **Kozak sequence generated** | **Primer sequence (5′ to 3′)** |
| --- | --- | --- |
| RP-Luc-177 |  | CATTTCGAAGTACTCAGCGTAAGTG |
| FP-AAAAAA | AAAAAatgA | CATTTCGAATAAAATCTCAAAAAAAATGAAAGACGCCAAAAAC |
| FP-AAAAAC | AAAAAatgC | CATTTCGAATAAAATCTCAAAAAAAATGCAAGACGCCAAAAAC |
| FP-AAAAAG | AAAAAatgG | CATTTCGAATAAAATCTCAAAAAAAATGGAAGACGCCAAAAAC |
| FP-TAAAAA | TAAAAatgA | CATTTCGAATAAAATCTCAATAAAAATGAAAGACGCCAAAAAC |
| FP-ACAAAA | ACAAAatgA | CATTTCGAATAAAATCTCAAACAAAATGAAAGACGCCAAAAAC |
| FP-AAACAC | AAACAatgC | CATTTCGAATAAAATCTCAAAAACAATGCAAGACGCCAAAAAC |
| FP-AAGAAA | AAGAAatgA | CATTTCGAATAAAATCTCAAAAGAAATGAAAGACGCCAAAAAC |
| FP-ACAAAC | ACAAAatgC | CATTTCGAATAAAATCTCAAACAAAATGCAAGACGCCAAAAAC |
| FP-TTTAAA | TTTAAatgA | CATTTCGAATAAAATCTCAATTTAAATGAAAGACGCCAAAAAC |
| FP-ACACAA | ACACAatgA | CATTTCGAATAAAATCTCAAACACAATGAAAGACGCCAAAAAC |
| FP-TTTTTA | TTTTTatgA | CATTTCGAATAAAATCTCAATTTTTATGAAAGACGCCAAAAAC |
| FP-CATCAA | CATCAatgA | CATTTCGAATAAAATCTCAACATCAATGAAAGACGCCAAAAAC |
| FP-AAACGA | AAACGatgA | CATTTCGAATAAAATCTCAAAAACGATGAAAGACGCCAAAAAC |
| FP-GAAGGG | GAAGGatgG | CATTTCGAATAAAATCTCAAGAAGGATGGAAGACGCCAAAAAC |
| FP-TCCTAC | TCCTAatgC | CATTTCGAATAAAATCTCAATCCTAATGCAAGACGCCAAAAAC |
| FP-AAGGGA | AAGGGatgA | CATTTCGAATAAAATCTCAAAAGGGATGAAAGACGCCAAAAAC |
| FP-CAAACA | CAAACatgA | CATTTCGAATAAAATCTCAACAAACATGAAAGACGCCAAAAAC |
| FP-GAAATC | GAAATatgC | CATTTCGAATAAAATCTCAAGAAATATGCAAGACGCCAAAAAC |
| FP-TTATAA | TTATAatgA | CATTTCGAATAAAATCTCAATTATAATGAAAGACGCCAAAAAC |
| FP-TCAAAA | TCAAAatgA | CATTTCGAATAAAATCTCAATCAAAATGAAAGACGCCAAAAAC |
