# Supplementary material for: Upstream AUGs and upstream ORFs can regulate the downstream ORF in Plasmodium falciparum
Source: Malar J. 2015 Dec 21;14:512. doi: 10.1186/s12936-015-1040-5 (PMC4687322; doi:10.1186/s12936-015-1040-5)
Supplement: Supplementary file 3 — 10.1186/s12936-015-1040-5 RT-PCR of the mixed stage parasite RNA. [file 12936_2015_1040_MOESM3_ESM.docx]

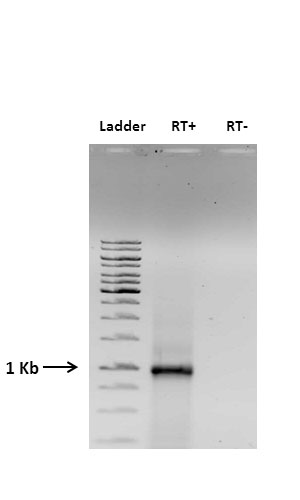


Additional figure 1 (Additional file 3). Agarose gel of the RT-PCR products of mixed stage parasite RNA. Expected size of the RT-PCR product is ~1kb (RT+). RT- reaction (without reverse transcriptase) was used as negative control. The primer used for RT-PCR reaction can amplify several *var* genes (21). For size comparison 1 kb step up DNA ladder (Thermo Scientific) was loaded.
